# Preoperative and postoperative imaging features in thoracic surgery: insights from a single-center study

**DOI:** 10.25122/jml-2025-0121

**Published:** 2025-08

**Authors:** Raluca Oltean, Liviu Oltean, Andreea Nelson Twakor, Teodor Horvat

**Affiliations:** 1 Carol Davila University of Medicine and Pharmacy, Bucharest, Romania; 2 Department of Internal Medicine, County Clinical Emergency Hospital, Constanta, Romania; 3 Department of Thoracic Surgery, Prof. Dr. Al. Trestioreanu Bucharest Oncological Institute, Bucharest, Romania

**Keywords:** thoracic surgery, preoperative imaging, radiologic risk stratification, ICU admission, postoperative complications

## Abstract

Thoracic surgery encompasses a broad spectrum of procedures with varying levels of risk. Preoperative imaging plays a critical role in evaluating anatomical pathology, but its predictive value for postoperative complications remains underexplored. This study aimed to assess whether specific radiologic features identified before surgery can predict key adverse outcomes, including ICU admission, in-hospital mortality, and length of hospital stay. We conducted a retrospective cohort study of 227 adult patients who underwent thoracic surgeries, including lobectomy, esophagectomy, thymectomy, and mediastinotomy, between 2019 and 2024. Preoperative imaging findings from chest radiographs, CT, PET-CT, MRI, and bronchoscopy were coded and analyzed. Outcomes included ICU admission, in-hospital mortality, and hospitalization duration. Univariate and multivariate logistic regressions were used to assess associations between imaging features and outcomes. Non-parametric tests and visual network plots were also applied. Common imaging findings included emphysema (29.1%), pleural effusion (12.8%), and nodules/metastases (7.9%). ICU admission occurred in 15% of patients, and in-hospital mortality occurred in 7.5%. Certain radiologic features, such as mediastinal lymphadenopathy (OR = 2.03) and nodules/metastases, showed a trend toward increased ICU admission. Conversely, features like bronchogram and no abnormalities were associated with a lower risk. Visual network analyses supported these trends. Preoperative imaging features, particularly those related to mediastinal or tumor burden, may offer predictive value for identifying patients at elevated postoperative risk. Incorporating radiologic markers into preoperative assessment could improve surgical planning and triage for intensive monitoring.

## INTRODUCTION

Thoracic surgery comprises a wide range of procedures, including pulmonary resections, esophagectomy, thymectomy, and mediastinal exploration. While advances in perioperative care and minimally invasive techniques have improved surgical outcomes, postoperative complications such as respiratory failure, prolonged hospitalization, and in-hospital mortality remain a clinical concern [1]. Early identification of high-risk patients is essential for optimizing surgical planning and postoperative management. Traditionally, risk stratification has relied on clinical parameters, functional respiratory testing, and tumor staging. However, the potential role of preoperative imaging findings in predicting postoperative outcomes has not been fully explored in the context of non-cardiac thoracic surgery [2].

**Figure 1:**
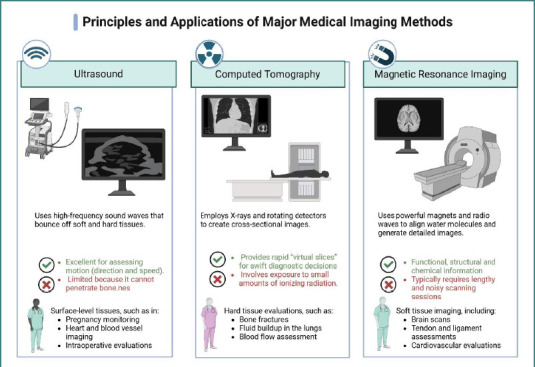
Medical imaging options. Created with Biorender [4]

Radiologic assessment is an integral part of preoperative evaluation. Imaging modalities such as chest radiography, computed tomography, PET-CT, and magnetic resonance imaging provide valuable information on pulmonary parenchyma, mediastinal anatomy, and tumor characteristics (Figure 1) [3,4].

Each technique has unique physical principles and clinical uses. High-frequency ultrasound is useful for superficial structural assessments such as pregnancy monitoring, cardiovascular imaging, and intraoperative assessments [5]. Visualizing structures behind the bone is limited. CT imaging uses X-rays and rotating detectors to create cross-sectional body images, making it helpful in detecting fluid accumulation and dense structures like bones. It uses little ionizing radiation but provides quick imaging [6]. However, MRI uses large magnets and radiofrequency waves to examine soft tissues like the brain, ligaments, and cardiovascular structures. It delivers good structural and functional data but is noisy and takes longer to scan [7].

Emphysema, pleural effusion, mediastinal lymphadenopathy, and tumor-related airway compression may signal more complex pathology, although risk prediction models rarely include these. Preoperative risk classification should be more nuanced and objective by identifying radiologic characteristics that correlate with postoperative ICU admission, death, or protracted hospitalisation [8].

This study evaluated how preoperative imaging features affect postoperative outcomes in a heterogeneous thoracic surgery group. We included individuals receiving lobectomy, esophagectomy, thymectomy, and mediastinotomy to expand our study beyond pulmonary resection [9]. We used statistical modelling and visual network analysis to assess the prognostic power of radiologic patterns for ICU admission, in-hospital mortality, and hospital stay duration. Imaging-based risk assessment may be integrated into surgical planning and perioperative decision-making based on our findings [10].

## MATERIAL AND METHODS

### Study design and population

This retrospective cohort study was conducted at a tertiary thoracic surgery center and included all adult patients (≥18 years) who underwent thoracic surgical procedures between January 2019 and December 2024 at Prof. Dr. Alexandru Trestioreanu Oncological Institute in Bucharest. The cohort included a diverse range of thoracic pathologies and surgical interventions. In addition to pulmonary resections (lobectomy, segmentectomy, wedge resection, pneumonectomy), the study population included patients who underwent esophagectomy, thymectomy, mediastinotomy, pleural decortication, and other chest wall or mediastinal procedures.

Patients were eligible for inclusion if they had complete preoperative imaging data and documented postoperative clinical outcomes. Exclusion criteria included incomplete imaging documentation or missing hospitalization outcome variables.

### Data collection and radiologic evaluation

Demographic and clinical data were extracted from electronic medical records, including age, sex, comorbidities, type of surgical intervention, and postoperative course. Preoperative and postoperative imaging data were collected from chest radiographs, thoracic computed tomography, magnetic resonance imaging, positron emission tomography, and bronchoscopy reports.

We considered the following definitions:

- Emphysema: defined on CT as areas of low attenuation (< -950 HU) without distinct walls, consistent with radiologic literature standards [11].
- Pleural effusion: defined as measurable pleural fluid accumulation on CT [12].
- Esophageal wall thickening: defined as circumferential or eccentric thickening >5 mm on axial CT slices [13].
- Ground-glass opacity: defined as hazy areas of increased lung attenuation without obscuration of underlying vessels [14].

For pulmonary resections (e.g., lobectomy, pneumonectomy), the primary radiologic features included:

- Emphysema
- Pleural effusion
- Atelectasis or pleural thickening
- Ground-glass opacities
- Pulmonary nodules or metastases
- Mediastinal lymphadenopathy
- Bronchogram pattern
- No radiologic abnormalities

For patients undergoing esophagectomy, the following additional features were recorded when present:

- Esophageal wall thickening
- Mediastinal lymphadenopathy
- Periesophageal fat infiltration
- Tracheoesophageal compression or deviation

For thymectomy and mediastinotomy cases, specific imaging markers included:

- Anterior mediastinal mass or soft tissue density
- Calcifications within thymic or mediastinal lesions
- Mediastinal lymphadenopathy
- Compression of adjacent mediastinal structures (vessels, airway)
- Signs of local invasion into the lung, pericardium, or major vessels

### Imaging acquisition and analysis

All features were recorded as binary variables (present or absent) based on standardized radiologic definitions. When available, radiologic reports were corroborated by independent review from board-certified radiologists who were blinded to the patients’ postoperative outcomes. This structured imaging approach was designed to capture both general thoracic abnormalities and procedure-specific features that may influence postoperative risk and recovery.

In this study, radiologic evaluation incorporated multiple imaging modalities routinely used in preoperative thoracic assessment. These included chest CT as the primary modality, supplemented by chest radiography, PET-CT, MRI, and bronchoscopy where clinically indicated. Imaging selection was based on patient-specific diagnostic requirements and institutional protocols. While thoracic CT served as the core imaging tool for most patients and was prioritized in the statistical analysis, relevant findings from other modalities were integrated when they contributed meaningful diagnostic information. The number and percentage of patients undergoing each modality are reported to ensure transparency. This multimodal approach reflects real-world clinical practice and was designed to capture the full spectrum of radiologic abnormalities relevant to perioperative risk stratification.

All radiologic features were evaluated as binary variables, classified as either present or absent. This approach was chosen to ensure consistency across varying imaging modalities and reporting styles. Emphysema was identified on computed tomography by the presence of well-demarcated areas of low attenuation without visible walls, in line with accepted radiologic criteria. Pleural effusion was defined by the visible accumulation of fluid within the pleural space, as seen on chest X-ray, CT, or ultrasound. Given the retrospective nature of the study and the variability in image acquisition protocols, quantitative or severity-based grading (e.g., volumetric analysis) was not feasible. However, all imaging data were reviewed by board-certified radiologists, and uncertain cases were resolved through consensus discussions with thoracic surgeons to ensure diagnostic accuracy. This binary classification allowed for systematic analysis while maintaining reproducibility across the cohort.

### Statistical analysis

For this research, we performed analyses using IBM SPSS Statistics version 27.0 (IBM Corp., Armonk, NY, USA) [15]. We used descriptive statistics to summarize patient characteristics, imaging findings, and outcomes. We reported categorical data as counts and percentages, and we expressed continuous variables as mean ± standard deviation or median and interquartile range, as appropriate. We also performed univariate logistic regression analyses to assess the association between individual imaging features and binary outcomes (ICU admission, mortality). Variables with a *P* value <0.10 in univariate analysis were included in multivariate logistic regression models to identify independent predictors. We also reported adjusted odds ratios (aORs) and 95% confidence intervals (CIs). All tests were two-tailed, and a *P* value <0.05 was considered statistically significant.

## RESULTS

### Population characteristics

This analysis covered 227 patients hospitalized between 2019 and 2024 in the thoracic surgery department. Lobectomy was performed in 125 patients (55.1%), followed by esophagectomy (18.5%), thymectomy (15.0%), and mediastinotomy (11.5%). The median age was 57, with a male predominance (56.4%) and a 65.2% urban population. Nearly 29% of patients smoked, and 28.2% were classified as obese [16].

The most common pre-existing conditions were hypertension (37.4%), diabetes (18.5%), and chronic obstructive pulmonary disease (COPD) (15.0%). Although an inflammation syndrome was present (C-reactive protein [CRP], erythrocyte sedimentation rate [ESR], and fibrinogen were slightly higher), preoperative laboratory results were within clinical ranges.

The most prevalent radiologic findings were emphysema (29.1%), pleural effusion (12.8%), atelectasis or thickening (15.0%), and suspicious nodules or metastases (7.9%). Esophageal wall thickening, mediastinal lymphadenopathy, and anterior mediastinal masses were added to the radiologic risk profile.

Table 1 presents a comprehensive summary of demographic, clinical, laboratory, imaging, and surgical data.

Many patients had clinically severe thoracic abnormalities before surgery, including emphysema (29.5%), pleural effusion (13.2%), and ground-glass infiltrates (12.8%). Together with procedure-specific characteristics, including esophageal wall thickening (7.0%) and mediastinal lymphadenopathy (11.5%), our data demonstrate the relevance of imaging in capturing disease complexity before surgery. During high-risk procedures like esophagectomy (18.5%) or mediastinotomy (11.4%), such radiologic characteristics may assist in stratifying patients at risk for problems.

**Table 1:** Population characteristics

Category	Variable	Mean ± SD or *n* (%)
**Demographics**	Age (years)	58.00 ± 9.95
	Male	130 (57.3%)
	Urban	150 (66.1%)
	Smoker	63 (27.8%)
	Obesity	66 (29.1%)
**Blood & Biochemistry**	Urea (mg/dL)	17.50 ± 5.30
	Creatinine (mg/dL)	0.91 ± 0.50
	TGO (U/L)	15.00 ± 2.3
	TGP (U/L)	26.50 ± 9.00
	Hemoglobin (g/dL)	13.40 ± 1.50
	WBC (10^3/µL)	7.60 ± 1.88
	Platelets (10^3/µL)	253.20 ± 61.80
	CRP (mg/L)	8.50 ± 4.90
	ESR (mm/h)	23.10 ± 10.00
	LDH (U/L)	190.00 ± 49.00
	Fibrinogen (g/L)	3.50 ± 0.70
**Imaging Findings**	No abnormalities	43 (18.9%)
	Emphysema	67 (29.5%)
	Atelectasis/pleural thickening	35 (15.4%)
	Interstitial changes	21 (9.3%)
	Drainage or pleural device	28 (12.3%)
	Alveolar infiltrates/ground-glass	29 (12.8%)
	Pleural effusion	30 (13.2%)
	Nodules/metastases	19 (8.4%)
	Esophageal wall thickening	16 (7.0%)
	Mediastinal lymphadenopathy	26 (11.5%)
	Anterior mediastinal mass	15 (6.6%)
	Tracheoesophageal compression	13 (5.7%)
	Invasion of adjacent structures	12 (5.3%)
**Comorbidities**	Hypertension	86 (37.9%)
	Diabetes	43 (18.9%)
	COPD	35 (15.4%)
	Chronic kidney disease	22 (9.7%)
	Heart failure	19 (8.4%)
	Atrial fibrillation	19 (8.4%)
	History of cancer	20 (8.8%)
**Surgical Details**	VATS lobectomy	79 (34.8%)
	Open lobectomy	46 (20%)
	Esophagectomy	42 (18.5%)
	Thymectomy	34 (14.9%)
	Mediastinotomy	26 (11.4%)
**Postoperative Management**	Postoperative drainage tube	196 (86.3%)
	Antibiotic therapy	167 (73.6%)
	Corticosteroid therapy	57 (25.1%)
	Oxygen therapy	75 (33.0%)
	Anticoagulation	33 (14.5%)
**Diagnostic Procedures**	Chest X-ray	211 (93.0%)
	Thoracic CT	203 (89.4%)
	Abdominal ultrasound	132 (58.1%)
	Cardiac ultrasound	94 (41.4%)
	PET-CT	38 (16.7%)
	Thoracic MRI	20 (8.8%)
	Bronchoscopy	27 (11.9%)
**Outcomes**	ICU admission	35 (15.4%)
	In-hospital mortality	18 (7.9%)
	Days of hospitalization	9.90 ± 4.00

Second, postoperative treatment and ICU utilization were comparable across the population despite clinical variability. No matter the pathology, most patients received standardized postoperative care, including chest drainage (86.3%), antibiotic medication (73.6%), and oxygen support (32.9%). The ICU admission (15.4%) and in-hospital death (7.9%) rates were also comparable to thoracic surgery populations.

### Predictive value of preoperative imaging features for ICU admission

We performed a univariate logistic regression in order to determine which preoperative imaging features predict postoperative intensive care [17] (Table 2). Emphysema, pleural effusion, and ground-glass opacities were included in the analysis, along with features relevant to esophagectomy, thymectomy, and mediastinotomy, such as esophageal wall thickening, mediastinal lymphadenopathy, and anterior mediastinal mass.

**Table 2:** Univariate logistic regression: preoperative imaging predictors of ICU admission

Predictor	OR	95% CI Lower	95% CI Upper	*P* value
No abnormalities	2.250639	0.092852	0.222548	0.04916
Emphysema	1.009411	0.114309	0.267378	0.982271
Pleural effusion	1.264870	0.111563	0.25209	0.61349
Atelectasis	1.710526	0.100103	0.236442	0.198641
Ground glass	0.370536	0.13204	0.281436	0.189665
Nodules	0.991304	0.119024	0.258591	0.987919
Esophageal wall thickening	0.947917	0.120757	0.256002	0.945844
Mediastinal lymphadenopathy	2.031635	0.927905	1.332661	0.092281
Anterio mediastinal mass	1.669642	0.117617	0.24897	0.534021
Tracheoesophageal compression	0.421911	0.125811	0.264212	0.413316
Invasion of adjacent structures	0.421911	0.125811	0.264212	0.413316

We assessed the odds ratio (OR), 95% confidence interval (CI), and statistical significance (*P* value) for each characteristic and ICU admission following surgery. An odds ratio above 1 implies a higher risk of ICU admission, while a ratio below 1 indicates a protective or neutral effect. *P* values below 0.05 show statistical significance, but those between 0.05 and 0.10 may imply clinical relevance [17].

Mediastinal lymphadenopathy on preoperative imaging was the best predictor (OR = 2.03; *P* = 0.092), suggesting that patients with this characteristic may need ICU care twice as often. Atelectasis and esophageal wall thickening also had higher ORs but were not statistically significant.

Most traits were not statistically significant, although a few showed promising tendencies.

For this statistical analysis, we performed a Kruskal-Wallis test, which is used for group comparison. Although the graphic does not display *P* values, it helps determine if imaging groups have meaningfully different ICU admission rates [18].

**Figure 2:**
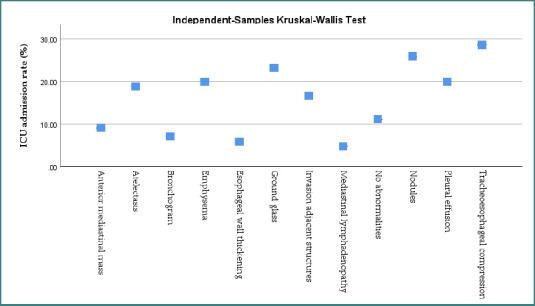
ICU admission rates by preoperative imaging feature (Kruskal-Wallis Test) [16]

Based on preoperative imaging, Figure 2 shows the frequency of ICU admissions after surgery. Each blue square shows the percentage ICU admission rate for an imaging finding like emphysema, nodules, or tracheo-esophageal compression.

According to the graph, some findings increase the likelihood of ICU admission. Patients with tracheo-esophageal compression or nodules had ICU admission rates exceeding 25%, indicating more complex or hazardous diseases. Patients with mediastinal lymphadenopathy, or no abnormalities, had considerably reduced ICU admission rates, typically below 10%.

**Figure 3:**
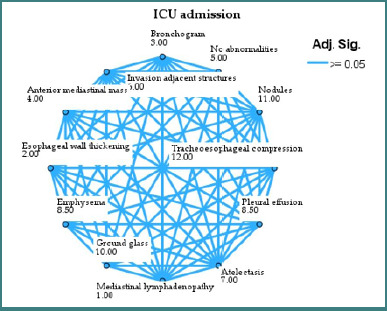
Network plot showing the relationship between imaging features and ICU outcome

**Figure 4:**
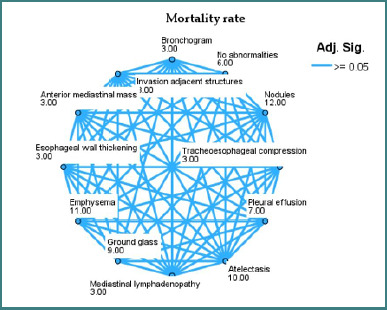
Network plot showing the relationship between imaging features and mortality

**Figure 5:**
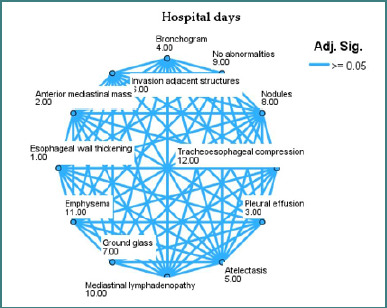
Network plot showing the relationship between imaging features and hospital days

The mean rank score from the non-parametric statistical comparison is shown in Figures 3-5 after each imaging finding. This rank shows how each characteristic affects the outcome variable [19].

More severe outcomes are related to higher-ranked nodes [20]. In the ICU admission plot, tracheo-esophageal compression (rank 12) and pleural effusion (rank 9) had higher average rankings, suggesting a stronger association with ICU need. Conversely, mediastinal lymphadenopathy (rank 1) and bronchogram (rank 3) were associated with decreased ICU utilization, suggesting lesser postoperative acuity.

The edges show no significant variations in rank scores between nodes (adjusted *P* > 0.05). In essence, a connecting line between two features indicates they have similar distributions for the outcome, whereas a lack of connection may indicate different clinical behaviour or outcome impact [21].

Ground-glass opacities, emphysema, and pleural effusion consistently rank high in all three areas, increasing the likelihood of prolonged hospitalisation or bad outcomes. No anomalies and mediastinal lymphadenopathy decrease rankings and improve postoperative outcomes.

These findings imply that radiologic abnormalities related to mediastinal and esophageal illness may indicate postoperative problems. Although more confirmation is needed, preoperative imaging may help stratify risk and plan perioperatively, especially in patients undergoing complex thoracic surgeries.

**Figure 6:**
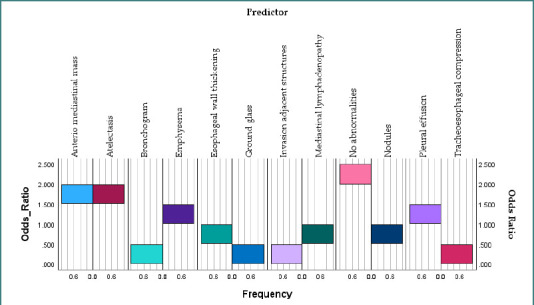
Odds ratios of preoperative imaging features for predicting ICU admission

In this analysis, bars above the value of 1 indicate an increased likelihood of ICU admission, while those below 1 suggest a lower or neutral risk (Figure 6).

Mediastinal lymphadenopathy and anterior mediastinal mass exhibited odds ratios around or exceeding 2.0, indicating that patients with these characteristics were twice as likely to need ICU treatment. Atelectasis, esophageal wall thickness, and pleural effusion were associated with higher chances, suggesting they may indicate surgical complexity or physiologic impairment. However, ground-glass opacities, bronchograms, and tracheo-esophageal compression were related to reduced risks of ICU admission, possibly due to less severe or localized pathology.

### Subgroup analysis: postoperative imaging findings in pulmonary lobectomy

To better explore the predictive and diagnostic role of imaging in a homogeneous surgical population, we conducted a focused subgroup analysis of the 125 patients who underwent pulmonary lobectomy. This group represented the largest procedural category in our cohort and allowed for direct comparison of pre- and postoperative imaging profiles.

The indications for pulmonary lobectomy in our cohort were predominantly oncologic. Specifically, 86% of patients underwent lobectomy for lung cancer, based on radiologic and histopathologic confirmation. The remaining 14% of cases involved benign but functionally significant pathology, including localized bronchiectasis, post-infectious fibrosis, or congenital malformations such as sequestration or cysts.

Post-lobectomy imaging revealed a clear reduction in pathological features commonly associated with thoracic surgical complexity. Emphysema decreased from 29.6% to 21.6%, pleural effusion from 12.8% to 3.2%, atelectasis or pleural thickening from 15.2% to 8.8%, and ground-glass infiltrates from 12.0% to 8.0%. The number of patients with no detectable radiologic abnormalities increased from 18.5% preoperatively to 24.8% postoperatively.

**Figure 7:**
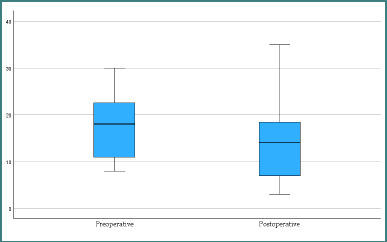
Distribution of imaging findings before and after pulmonary lobectomy

**Figure 8:**
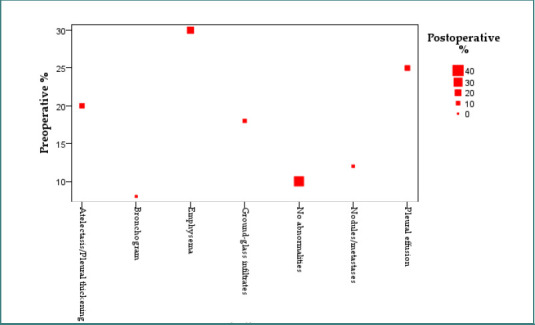
Bubble plot comparing preoperative and postoperative imaging findings

These findings are visually represented in Figures 7 and 8, where preoperative imaging showed a broader distribution of abnormalities, while postoperative imaging demonstrated convergence toward normal radiologic profiles.

In contrast, the postoperative group demonstrates a clear reduction in both the number and variability of findings, with a lower median and compressed IQR, indicating clinical and radiologic improvement after surgery.

Chest radiography (92%) and thoracic CT (89.6%) were the most frequently used modalities postoperatively. CT imaging proved superior in detecting subtle parenchymal changes like nodules, emphysema, and alveolar infiltrates. Table 3 summarizes the distribution of radiologic findings by modality, with thoracic CT detecting emphysema in 7.2% and nodular/metastatic lesions in 2.4% postoperatively.

**Table 3:** Comparison of preoperative imaging features with ICU admission, mortality, and hospital stay

Imaging feature preoperatively	ICU admission rate	Mortality rate	Mean hospital stay (days)
No abnormalities	11.10%	4.40%	10.86
Emphysema	20.00%	10.80%	11.63
Pleural effusion	20.00%	5.00%	9.93
Atelectasis	18.80%	10.40%	10.38
Ground glass	23.30%	10.00%	10.64
Nodules	25.90%	11.10%	10.82
Esophageal wall thickening	5.90%	0.00%	8.21
Mediastinal lymphadenopathy	4.80%	0.00%	11.08
Anterior mediastinal mass	9.10%	0.00%	9.68
Tracheoesophageal compression	28.60%	0.00%	11.94
Invasion of adjacent structures	16.70%	8.30%	10.43

In this subgroup, the ICU admission rate was 15.2%, and in-hospital mortality was 7.2%, aligning with broader trends observed across the entire thoracic surgery cohort. A Kaplan–Meier analysis [22] revealed similar early postoperative survival between open and video-assisted thoracoscopic surgery (VATS) approaches, though a slight divergence was noted in late-stage cumulative hazard estimates, with open lobectomy showing a steeper increase after postoperative day 12. The mean survival time was slightly higher in the open group (19.91 days) versus the VATS group (17.74 days), although this may reflect baseline differences and should be interpreted cautiously.

**Figure 9:**
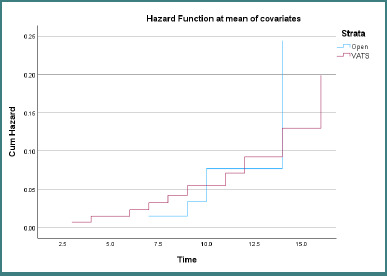
Cumulative hazard function comparing open vs. VATS lobectomy

The estimated mean survival time for the entire cohort was 19.29 days (95% CI, 17.76–20.83), with noticeable variation between surgical techniques (Figure 9). Patients who underwent open lobectomy had a higher mean survival estimate of 19.91 days (95% CI, 18.25–21.58), while the VATS group showed a slightly lower mean survival of 17.74 days (95% CI, 16.95–18.52). The median survival time could not be computed for the overall cohort or the open group due to right-censoring; however, the VATS group exhibited a median survival of 19 days.

## DISCUSSION

This study shows that preoperative imaging characteristics can predict thoracic surgery results. We found that mediastinal lymphadenopathy, nodules/metastases, and tracheo-esophageal compression increase ICU admission and in-hospital mortality. These findings support literature emphasizing comprehensive imaging assessments in surgical risk classification [23,24].

In 11% of our group, mediastinal lymphadenopathy was associated with increased ICU hospitalizations (OR = 2.03; *P* = 0.092). Zheng *et al*. found that enlarged mediastinal lymph nodes, especially those over 2.5 cm, are more likely to be malignant and have poor outcomes [25]. Baram *et al*. found that pneumonectomy patients with mediastinal lymphadenopathy had higher ICU admission and mortality rates [26].

The presence of nodules/metastases on preoperative imaging also predicted postoperative problems. These patients had the greatest ICU admission rates (25.9%) and mortality (11.1%). These findings support Leivaditis *et al*.'s finding that large anterior mediastinal masses increase perioperative risks, requiring careful preoperative planning [27].

Though rare, tracheo-esophageal compression increased ICU admissions. Borgheresi *et al*. [28] noted the intricacy of mediastinal anatomy and its complications in thoracic surgery. Imaging features like bronchograms and no abnormalities were associated with lower ICU admission and mortality rates, indicating a better prognosis. As Brady *et al*. found, the lack of major radiologic abnormalities reduces surgical complications [29].

Our study's strength is its detailed investigation of lobectomy, esophagectomy, thymectomy, and mediastinotomy. By integrating many imaging features, we can better comprehend their prognostic effects. However, the retrospective design and single-center arrangement may limit generalizability. Therefore, preoperative imaging features can predict postoperative ICU admission and mortality in thoracic surgery. These radiologic markers help improve risk stratification, surgical planning, and patient outcomes in preoperative examinations.

### Limitations

This retrospective, single-center study's findings may not be generalizable to other populations or institutions employing distinct surgical protocols or imaging methodologies. All patients received standard postoperative treatment; nevertheless, the time and modality of imaging may influence the detection and classification of radiologic findings.

Another limitation of this study is the heterogeneity in imaging modalities used across the cohort. Although thoracic CT was the most consistently available and served as the principal source for analytical comparisons, additional findings were derived from chest radiography, PET-CT, MRI, and bronchoscopy depending on clinical indications. This variability may introduce interpretive challenges and limit uniformity in radiologic assessment. However, this reflects routine multidisciplinary preoperative workflows in thoracic surgery, where multimodal imaging is often necessary for comprehensive evaluation.

One methodological limitation is the use of univariate logistic regression for preliminary variable selection in the multivariable models. This approach has been debated in the literature; however, it was deemed appropriate in our context due to the limited number of outcome events, particularly for ICU admission and in-hospital mortality. Adhering to the recommended events-per-variable ratio of at least 10 to avoid overfitting, our capacity to include multiple predictors in multivariable models was constrained. Univariate screening allowed us to identify candidate variables with potential relevance while maintaining model parsimony and stability. Although more advanced selection techniques (e.g., penalized regression or full-model strategies) may offer advantages in larger datasets, our chosen method reflects a pragmatic balance between statistical rigor and clinical interpretability, given the retrospective nature and sample size of the study.

Also, radiologic findings have previously been associated with postoperative complications. Our study aimed to systematically evaluate their predictive role within a structured imaging framework across a broad thoracic surgical population. Importantly, this work sought to explore whether radiological features could serve as early markers of clinical deterioration, complementing traditional clinical and physiological risk indicators. While the comparison between imaging findings and functional parameters such as pulmonary function tests (PFTs) would have been of high clinical relevance, this was not feasible in the present analysis due to incomplete documentation of formal metrics like forced expiratory volume in one second (FEV_1_) and diffusing capacity of the lungs for carbon monoxide (DLCO) in the retrospective dataset. As a result, PFTs could not be incorporated into the statistical modelling. This represents a limitation of the current work. Nevertheless, our findings highlight the independent contribution of imaging to risk stratification, and we propose that future prospective studies should integrate radiologic and physiologic data to better define their combined prognostic value in thoracic surgery.

The absence of long-term postoperative imaging constitutes an additional limitation. Subsequent issues or alterations in recurrence patterns may have been overlooked throughout the investigation. Functional respiratory metrics were not evaluated in conjunction with imaging results, complicating the correlation between radiologic enhancement and physiological recovery.

## CONCLUSION

The study shows that preoperative imaging features can predict short-term postoperative outcomes in thoracic surgery patients. We detected radiologic abnormalities associated with increased risk of ICU admission, in-hospital mortality, and prolonged hospitalisation in 227 patients who underwent lobectomy, esophagectomy, thymectomy, and mediastinotomy.

Mediastinal lymphadenopathy, found in 11% of patients, was related to a nearly twofold increase in ICU admission (OR = 2.03; *P* = 0.092), suggesting it may be a high-risk marker. Tumour load may be linked to postoperative decompensation, as nodules or suspected metastases had the highest ICU admission rate (25.9%) and fatality rate (11.1%). Emphysema, pleural effusion, and atelectasis also caused longer hospital stays and increased critical care use.

However, imaging markers including bronchograms and absence of abnormalities were related to decreased complication rates, indicating a better risk profile. Imaging helps stratify risk early, as patients lacking radiologic abnormalities had shorter hospital stays, reduced ICU admission, and fatality rates.

We conclude that the radiologic profile before thoracic surgery gives prognostic information. Mediastinal lymphadenopathy, tracheo-esophageal compression, and tumours may indicate postoperative difficulty. Adding these imaging variables to preoperative assessments could improve surgical planning, targeted monitoring, and patient outcomes. Radiologic risk ratings and predictors need further prospective research to improve and validate in larger surgical populations.
